# Supplementary material for: Complexome profiling of the Chlamydomonas psb28 mutant reveals TEF5 as an early PSII assembly factor
Source: Plant Cell. 2025 Mar 17;37(6):koaf055. doi: 10.1093/plcell/koaf055 (PMC12164590; doi:10.1093/plcell/koaf055)
Supplement: koaf055_Supplementary_Data [file koaf055_supplementary_data.zip › psb28_interactive.zip!/plots/intensity/qualityPlots/peptides/tufA_Cre-1.g2716979.t1.p1.html]

### Cre-1.g2716979.t1.p1

Trivial name: tufA  
SeqCoverage [median]: 23.90  
Mass: 45.74 kDa  
  
 

| Sequence | MissedCleavage | Mass [Da] | wtIntensity | muIntensity | Score |
| --- | --- | --- | --- | --- | --- |
| GITINTAHVEYETEKR | 1 | 1859.94 | 665.20 | 0.00 | 42.95 |
| ISMTVELINPIAIEK | 0 | 1669.93 | 524.88 | 0.00 | 87.36 |
| KYDEIDSAPEEK | 1 | 1422.65 | 730.20 | 581.00 | 71.89 |
| NPSSVAEEHSNK | 0 | 1297.59 | 2171.91 | 0.00 | 50.46 |
| TLDETLAGDNVGVLLR | 0 | 1684.90 | 1021.27 | 0.00 | 130.56 |
| TVGAGVVTNIVQ | 0 | 1156.65 | 933.96 | 1236.78 | 78.33 |
| VVGFNHIQMR | 0 | 1199.62 | 668.83 | 546.10 | 46.89 |
|  |  |  | 6716.2 | 2363.9 |  |
